# Respiratory and gastrointestinal symptoms in esophageal atresia

**DOI:** 10.1186/1824-7288-34-5

**Published:** 2008-12-08

**Authors:** Fernando Maria de Benedictis, Ascanio Martino

**Affiliations:** 1 Division of Pediatrics, AziendaOspealiero-Universitaria "Ospedali Riuniti", Ancona, Italy; 2 Division of Pediatric Surgery, AziendaOspealiero-Universitaria "Ospedali Riuniti", Ancona, Italy

Sir,

Mastroianni et al. [1] report high prevalence (100%) of respiratory symptoms in their cohort of 19 children who received surgery for esophageal atresia. In contrast with the results of other studies [2], they found gastro-esophageal reflux incidence was low (26,3%). They therefore emphasize anti-reflux therapy could not be effective for treatment of respiratory symptoms in this group of patients.

We agree with the speculation of the authors, as this was also our experience. Indeed, children who received surgical intervention for esophageal atresia have high incidence of respiratory function abnormalities [3] and bronchial hyperreactivity [4]. These conditions may be multifactorial in origin and are related to recurrent inhalation, epithelial damage, bronchial obstruction and tracheomalacia.

Treatment of underlying respiratory disease instead of anti-reflux therapy may be warranted in children with a history of surgical repair of esophageal atresia and tracheoesophageal fistula, especially when lung function studies confirm respiratory involvement.
